# Cefiderocol resistance genes identified in environmental samples using functional metagenomics

**DOI:** 10.1093/ismejo/wrag010

**Published:** 2026-01-28

**Authors:** Remi Gschwind, Mehdi Bonnet, Anna Abramova, Victor Hugo Jarquín-Díaz, Marcus Wenne, Ulrike Löber, Nicolas Godron, Ioannis D Kampouris, Faina Tskhay, Fouzia Nahid, Chloé Debroucker, Maximilien Bui-Hai, Inès El Aiba, Uli Klümper, Thomas U Berendonk, Sofia K Forslund-Startceva, Rabaab Zahra, Johan Bengtsson-Palme, Etienne Ruppé

**Affiliations:** Université Paris Cité and Université Sorbonne Paris Nord, INSERM, IAME, 16 rue Henri Huchard, Paris F-75018, France; Université Paris Cité and Université Sorbonne Paris Nord, INSERM, IAME, 16 rue Henri Huchard, Paris F-75018, France; AP-HP, Hôpital Beaujon, Laboratoire de Bactériologie, 100 Boulevard du Général Leclerc, Clichy F-92110, France; Department of Infectious Diseases, Institute of Biomedicine The Sahlgrenska Academy, University of Gothenburg, Guldhedsgatan 10, Gothenburg SE-413 46, Sweden; Division of Systems and Synthetic Biology, Department of Life Sciences, SciLifeLab, Chalmers University of Technology, Chalmersplatsen 1, Gothenburg SE-412 96, Sweden; Centre for Antibiotic Resistance Research (CARe), Guldhedsgatan 10, Gothenburg SE-412 96, Sweden; Experimental and Clinical Research Center, a cooperation between the Max-Delbrück-Center for Molecular Medicine in the Helmholtz Association and the Charité - Universitätsmedizin Berlin, Charitépl. 1, 10117 Berlin, Germany; Max Delbrück Center for Molecular Medicine in the Helmholtz Association (MDC), Robert-Rössle-Straße 10, 13125 Berlin, Germany; Charité-Universitätsmedizin Berlin, Freie Universität Berlin and Humboldt-Universität zu Berlin, Unter den Linden 6, 10117 Berlin, Germany; DZHK, German Centre for Cardiovascular Research, Potsdamer Str. 58, 10785 Berlin, Germany; Department of Infectious Diseases, Institute of Biomedicine The Sahlgrenska Academy, University of Gothenburg, Guldhedsgatan 10, Gothenburg SE-413 46, Sweden; Division of Systems and Synthetic Biology, Department of Life Sciences, SciLifeLab, Chalmers University of Technology, Chalmersplatsen 1, Gothenburg SE-412 96, Sweden; Experimental and Clinical Research Center, a cooperation between the Max-Delbrück-Center for Molecular Medicine in the Helmholtz Association and the Charité - Universitätsmedizin Berlin, Charitépl. 1, 10117 Berlin, Germany; Max Delbrück Center for Molecular Medicine in the Helmholtz Association (MDC), Robert-Rössle-Straße 10, 13125 Berlin, Germany; Charité-Universitätsmedizin Berlin, Freie Universität Berlin and Humboldt-Universität zu Berlin, Unter den Linden 6, 10117 Berlin, Germany; DZHK, German Centre for Cardiovascular Research, Potsdamer Str. 58, 10785 Berlin, Germany; Université Paris Cité and Université Sorbonne Paris Nord, INSERM, IAME, 16 rue Henri Huchard, Paris F-75018, France; Technische Universität Dresden, Institute of Hydrobiology, Zellescher Weg 40, Dresden, 01217, Germany; Technische Universität Dresden, Institute of Hydrobiology, Zellescher Weg 40, Dresden, 01217, Germany; Department of Microbiology, Faculty of Biological Sciences, Quaid-i-Azam University, University Rd, Islamabad, Pakistan; Université Paris Cité and Université Sorbonne Paris Nord, INSERM, IAME, 16 rue Henri Huchard, Paris F-75018, France; Université Paris Cité and Université Sorbonne Paris Nord, INSERM, IAME, 16 rue Henri Huchard, Paris F-75018, France; Université Paris Cité and Université Sorbonne Paris Nord, INSERM, IAME, 16 rue Henri Huchard, Paris F-75018, France; Technische Universität Dresden, Institute of Hydrobiology, Zellescher Weg 40, Dresden, 01217, Germany; Technische Universität Dresden, Institute of Hydrobiology, Zellescher Weg 40, Dresden, 01217, Germany; Experimental and Clinical Research Center, a cooperation between the Max-Delbrück-Center for Molecular Medicine in the Helmholtz Association and the Charité - Universitätsmedizin Berlin, Charitépl. 1, 10117 Berlin, Germany; Max Delbrück Center for Molecular Medicine in the Helmholtz Association (MDC), Robert-Rössle-Straße 10, 13125 Berlin, Germany; Charité-Universitätsmedizin Berlin, Freie Universität Berlin and Humboldt-Universität zu Berlin, Unter den Linden 6, 10117 Berlin, Germany; DZHK, German Centre for Cardiovascular Research, Potsdamer Str. 58, 10785 Berlin, Germany; Structural and Computational Biology Unit, EMBL, Meyerhofstraße 1, 69117 Heidelberg, Germany; Department of Microbiology, Faculty of Biological Sciences, Quaid-i-Azam University, University Rd, Islamabad, Pakistan; Department of Infectious Diseases, Institute of Biomedicine The Sahlgrenska Academy, University of Gothenburg, Guldhedsgatan 10, Gothenburg SE-413 46, Sweden; Division of Systems and Synthetic Biology, Department of Life Sciences, SciLifeLab, Chalmers University of Technology, Chalmersplatsen 1, Gothenburg SE-412 96, Sweden; Centre for Antibiotic Resistance Research (CARe), Guldhedsgatan 10, Gothenburg SE-412 96, Sweden; Université Paris Cité and Université Sorbonne Paris Nord, INSERM, IAME, 16 rue Henri Huchard, Paris F-75018, France; AP-HP, Hôpital Bichat-Claude Bernard, Laboratoire de Bactériologie, 46 Rue Henri Huchard, 75018 Paris, France

**Keywords:** cefiderocol, antibiotic resistance, functional metagenomics

## Abstract

Antibiotic resistance poses a global public health threat, which can originate from the transfer of environmental antibiotic resistance genes to pathogenic bacteria, as highlighted by the “One Health” framework. Cefiderocol is a siderophore cephalosporin recently introduced in clinical practice which displays a “Trojan Horse” mechanism, utilizing bacterial iron transportation systems for cell entry. Although it is only used as a last-line antibiotic, resistance has already been observed in clinical isolates. Yet, cefiderocol resistance genes are difficult to monitor as resistance mechanisms remain mostly undescribed in antibiotic resistance gene databases and therefore uncharacterized in the environment. To address this critical gap, we applied functional metagenomics to diverse environmental samples (wastewater, freshwater, and soil) from France, Germany, Sweden, and Pakistan. Four antibiotic resistant genes were identified as responsible for increased cefiderocol minimum inhibitory concentrations to clinically-relevant levels (ranging from 1 to 4 mg/l), including ꞵ-lactamases (VEB-3, OXA-372 homolog, and YbxI homolog) and a partial penicillin-binding protein homolog. None of these genes had been previously reported as a cefiderocol resistance gene. Three out of four had their closest homologs in pathogenic bacteria. The *bla*_VEB-3_ gene was associated with a mobile genetic element and distributed across all wastewater metagenomes analyzed in this study. We therefore highlight the critical need for functional metagenomics, to characterize previously uncharacterized last-line antibiotic resistance mechanisms which will be used to enrich antibiotic resistance gene databases and thereby improving antibiotic resistance surveillance in all One Health compartments.

## Introduction

Antibiotic resistance represents a global public health threat, with resistant bacterial strains found in humans, animals, and the environment. This observation reinforces the “One Health” concept, which recognizes the interconnectedness of these compartments [1]. Beyond the dissemination of resistant strains, bacteria can also transfer or acquire genetic material through horizontal gene transfer. The acquisition of antibiotic resistance genes (ARGs) can lead to the emergence of difficult-to-treat multidrug-resistant bacteria which were associated with 4.71 million deaths worldwide in 2021 [2]. In 2024, the World Health Organization classified three extended-spectrum ꞵ-lactamase (ESBL)-producing and seven carbapenemase-producing *Enterobacterales* among its 24 priority pathogens, due to their increasing resistance to last line antibiotics and widespread dissemination [3, 4]. In this context, the development of new therapeutic solutions is crucial. Cefiderocol, a recently developed antibiotic approved for clinical use, is a cephalosporin bearing a catechol group which acts as a siderophore by forming chelated complexes with ferric iron [5]. Siderophores are molecules naturally produced by bacteria and secreted extracellularly to chelate iron. Employing a “Trojan Horse” strategy, iron-chelated cefiderocol utilizes the bacterial iron transport system to gain entry into the periplasm, where it disrupts cell wall biosynthesis by binding penicillin-binding proteins (PBPs).

Cefiderocol has demonstrated efficacy against a broad spectrum of Gram-negative bacterial isolates, including ESBL and carbapenemase producers [6–9]. Its stability against hydrolysis by class D ꞵ-lactamases (OXA-48, OXA-40, OXA-23) and other class A and class B ꞵ-lactamases (IMP-1, VIM-2, NDM-1, KPC-2, KPC-3, L1, VEB-1) has been established [10–12]. Cefiderocol-resistant isolates have been detected, even in patients without prior exposure to the antibiotic [13]. Cloning and expression studies have shown that certain ꞵ-lactamases, including class A (KPC, PER, SHV, BEL), class B (NDM), class C (AmpC), and class D (OXA) enzymes, are associated with reduced cefiderocol susceptibility [12, 14–19]. This can be attributed to the ability of these ꞵ-lactamases to hydrolyze or trap cefiderocol, as observed with some KPC variants [20]. Other resistance mechanisms involve target modifications. For instance, YRIN or YRIK insertions at position 338 in the PBP-3 encoding gene have been linked to elevated cefiderocol minimum inhibitory concentrations (MIC) [8, 13, 21, 22]. Membrane permeability modifications can also influence cefiderocol MICs [16]. Furthermore, mutations or deletions in genes involved in iron uptake can impact the cefiderocol resistance phenotype [8, 9, 13, 21, 23–28]. Cefiderocol resistance is associated not only with gene mutations or truncations but also with variations in gene expression [29]. This multifactorial nature of cefiderocol resistance complicates its study, making cloning and expression experiments the gold standard for elucidating the contribution of individual mechanisms.

Current research efforts are increasingly directed towards improving our understanding and monitoring strategies of ARGs [30]. Metagenomics facilitates the detection of ARGs within an environment or a bacterial strain genome by comparing sequences to ARG databases. However, ARG databases are not exhaustive, lacking data on environmental ARGs. Moreover, phenotypic data on the effect of ARGs on new antibiotics, such as cefiderocol, is also often limited. Functional metagenomics can address these gaps by identifying ARGs not yet described in existing databases or by providing phenotypic data for known ARGs [31]. This technique identifies ARGs based on phenotype rather than solely on sequence homology. It is performed by expressing DNA libraries in antibiotic-sensitive hosts in the presence of antibiotics. Resistant clones harbor a DNA fragment containing an ARG. Its sequence can be determined to increment ARG databases like ResFinderFG, a database of ARGs identified by functional metagenomics [32]. Furthermore, the resistant clones can be used to precisely characterize the associated resistance phenotype across an entire antibiotic family.

Cefiderocol resistance has been primarily studied in clinical strains, data regarding its prevalence in the environment remain scarce. Nonetheless, the environment is a crucial compartment within the One Health framework. Bacteria and their ARGs are transferred between compartments. The environment, characterized by its extensive niche diversity, harbors a highly diverse reservoir of genes including ARGs [33]. Environmental bacteria are considered the ancestral hosts for most ARGs, predating clinical antibiotic use, and the environment still serves as a shared source of ARGs to both environmental and pathogenic strains [34, 35]. In this study, we aimed to employ functional metagenomics to identify cefiderocol resistance genes across diverse environmental samples and subsequently study their dissemination.

## Materials and methods

### Sample collection and DNA isolation

Soil, wastewater, fish mucus, and freshwater samples were collected in Germany, Sweden, Pakistan and France between 2021 and 2022. DNA was extracted using the PowerSoil Pro DNA isolation kit (Qiagen, Hilden, Germany). Detailed methods for each sample type can be found in Supplementary Data. The quantity and quality of the extracted DNA were assessed using Qubit Broad Range assay along with Nanodrop measurements (Thermo Fisher, Waltham, USA).

### Metagenomic sequencing and microbial community characterization

Samples were sequenced by the SNP&SEQ Platform at the National Genomics Infrastructure, Uppsala, Sweden. Sequencing libraries were generated using the SMARTer thruPLEX DNA-seq kit (Takara Bio, Shiga, Japan). A total of 47 libraries were pooled across two lanes of a S4 flowcell and were sequenced using the NovaSeq 6000 System (Illumina, San Diego, USA). Taxonomic profiling of each metagenome was performed using mOTUs v3.1 [36]. Principal Coordinates Analysis (PCoA) was conducted based on Bray–Curtis dissimilarity between samples. Metagenomic contigs were assembled using NGLess v1.5 and MEGAHIT [37, 38].

### Functional metagenomic libraries and clone selection

Samples with at least 800 ng of remaining DNA post-sequencing were selected for functional metagenomics detection of cefiderocol resistance genes. DNA was sheared using the tagmentase enzyme from the Nextera XT kit (Illumina) to achieve a target size of 1–3 kb with an optimized protocol (Supplementary Data). The tagmented DNA was amplified and cloned in a pHSG299 expression vector (Takara Bio, Shiga, Japan; accession number: M19415; Fig. S1). Recombining plasmid was transformed into competent *Escherichia coli* K12 MG1655 susceptible to cefiderocol. Clones associated with an increased cefiderocol MIC were then selected on Luria-Bertani (LB) agar media supplemented with 100 mM IPTG and 1 mg/l cefiderocol to select for significant MIC increase and to avoid potential false positives associated with the use of low antibiotic concentrations. The use of iron-depleted media was not required as iron is sufficiently bound to agar, mimicking an iron-depleted state [39].

### Confirmation of plasmid mediated cefiderocol resistance

Confirmation of the cefiderocol-resistant phenotype was achieved by sequencing each clone and assembly of their genomic DNA to identify potential mutations in genes previously associated with cefiderocol resistance (Table S1) and by transforming fresh competent *E. coli* K12 MG1655 with the previously extracted plasmid containing the ARG bearing insert (Supplementary Data).

### Phenotypic characterization

Disc diffusion assays were performed in triplicates using MH agar media to assess susceptibility to a range of ꞵ-lactam antibiotics and ꞵ-lactam-ꞵ-lactamase inhibitor combinations (Supplementary Data). The interpretation of the disk diffusion assays and MIC determinations were strictly performed using the most recent (2025) breakpoints published by the European Committee on Antimicrobial Susceptibility Testing (EUCAST). MICs were determined in triplicates using iron-depleted cation-adjusted Mueller Hinton (CAMH) broth (Biocentric, France) following the EUCAST recommendations. Cefiderocol MICs were determined using unitary UMIC Cefiderocol tests (Bruker, Billerica, USA). To further investigate ꞵ-lactamase mediated resistance or the potential effect of the ꞵ-lactamase inhibitor, a nitrocefin hydrolysis test was performed along with cefiderocol-avibactam (4 mg/l) MICs determination in triplicates using unitary UMIC Cefiderocol tests (Bruker, Billerica, USA) in iron-depleted CAMH broth. To assess the intrinsic effect of avibactam alone, avibactam MICs were determined in triplicates in CAMH broth (Supplementary Data).

### Molecular characterization

The insert amplified via PCR was Sanger sequenced. Taxonomy of the insert sequence was determined using BLASTN against the NCBI nt database. ORFs within the insert were identified using PROKKA v1.14 and BAKTA webserver v1.11.0 annotation software [40, 41]. Cartography of each insert was made using clinker v0.0.21 [42]. Predicted ARG sequences from the insert were subjected to BLASTN analysis against ARG databases such as ResFinder 4.6.0 and ResFinderFG v2.0 [32, 43]. BLASTP was also used to identify protein variants within the NCBI protein database nr. The protein structure was predicted using AlphaFold 3 and structure alignments were performed using PyMOL (v3.1.4), both with default parameters [44, 45]. MatchAlign score divided by the sum of both protein lengths was used to compare alignments. ChimeraX software (v1.9) and the matchmaker function were used for structure alignment visualization [46]. Phylogenetic analysis was performed using previously functionally verified ꞵ-lactamase encoding genes [14]. Sequences were aligned using the EMBL-EBI web version of Clustal Omega and phylogenetic trees were built using IQ-TREE v2.2.0 [47], with ModelFinder (Supplementary Data). To study the insert environment, the genes identified were searched in metagenomic contigs obtained from metagenomic assemblies of each sample using BLASTN.

### Distribution of cefiderocol-resistant genes

A multi-step bioinformatic approach was employed to determine the environmental distribution and host species of cefiderocol resistance genes identified through functional metagenomics. First, to assess their relative abundance within several environments, metagenomic reads from each sample were mapped against the ARGs using Bowtie2. Next, homologous sequences were identified within the GMGC v1.0 [48], using a BLAST-like sequence similarity search on GMGC website (https://gmgc.embl.de/; queried on 28 April, 2025). Finally, BLASTN searches against the EnteroBase database were conducted to identify *E. coli* strains harboring these resistance genes [49].

## Results

### Functional metagenomics libraries production and selection of clones with increased cefiderocol MIC

**Figure 1:**
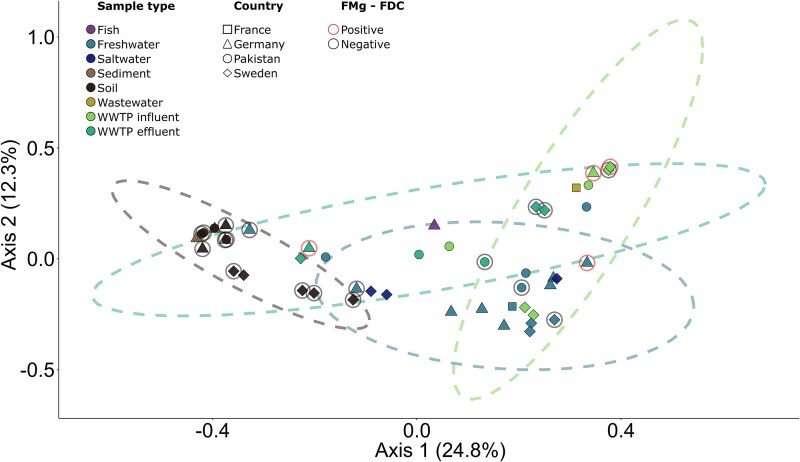
PCoA of the bacterial composition of each sequenced sample based on Bray–Curtis dissimilarity. Samples are classified based on their type, country of origin, whether they were analyzed with functional metagenomics, and whether they were positive or negative for detection of a clone with an increased cefiderocol MIC. Data ellipses were computed for sample types with enough data points (i.e. freshwater, soil, WWTP influent and WWTP effluent) using the function stat_ellipse() from ggplot2, with default statistical parameters (assuming multivariate t-distributions). WWTP: wastewater treatment plant; FMg-FDC: samples for which cefiderocol resistance was studied using functional metagenomics.

A total of 47 samples were collected for the EMBARK project (Table 1): 17 from Sweden, two from France, 16 from Germany, and 12 from Pakistan. All 47 samples were subjected to metagenomic sequencing, and 21 were selected for functional metagenomics analysis (Fig. S2). Four clones containing recombining DNA from four samples were selected on cefiderocol-containing media. These included three wastewater samples (SWE-1-JRYAIN, GER-1-KREISCHAIN, and GER-5-KREISCHAOUT) and one freshwater sample (GER-3-ELBEWATER). The increased cefiderocol MIC of each clone was confirmed to be due solely to the expression of the cloned DNA fragment, which contained a cefiderocol resistance gene (Supplementary Data and Table S2). Although samples were effectively differentiated by their environmental origin (wastewater influentor effluent, soil, and freshwater), those in which a cefiderocol resistance gene was identified by functional metagenomics did not cluster within any specific environmental category based on their bacterial community composition (Fig. 1).

**Table 1:** Environmental samples (n = 47) included in the EMBARK project.

**Country**	**Sample ID**	**Sample type**	**Input use for extraction**	**Date collected**	**Location**	**Coordinates**	**DNA concen-tration (ng/μL)**
Sweden	**SWE-1-JRYAIN**	**Influent**	**500** **ml, filtered**	**28.06.21**	**Gothenburg Ryaverket WWTP**	**57.6972, 1.8901**	**86.5**
	SWE-2-JFINN	Freshwater	6 l, filtered	29.06.21	Finnsjön drinking water lake	57.6339, 12.1492	18.6
	SWE-3-JRYAOUT	Effluent	2 l, filtered	28.06.21	Gothenburg Ryaverket WWTP	57.6972, 11.8901	40.4
	**SWE-4-JSOIL1**	**Soil**	**250 mg**	**29.06.21**	**Greggered pasture**	**57.6085, 12.1529**	**243.5**
	SWE-5-JGOTA	Freshwater	4.5 l filtered	30.06.21	Göta Älv river downstream	57.6907, 11.9058	40.4
	**SWE-6-JGOTAMP**	**Freshwater**	**1 l filtered**	**30.06.21**	**Göta Älv river downstream**	**57.6907, 11.9058**	**58.5**
	SWE-7-JFOTO	Saltwater	5 l filtered	30.06.21	Fotö sea water	57.6707, 11.6624	15.3
	SWE-8-JFOTO2	Saltwater	2.5 l filtered	30.06.21	Fotö sea water	57.6707, 11.6624	8.1
	SWE-9-NFOTO	Saltwater	3.5 l filtered	22.11.21	Fotö sea water	57.6707, 11.6624	46
	SWE-10-NGOTA	Freshwater	1.3 l filtered	22.11.21	Göta Älv river downstream	57.6907, 11.9058	65.8
	**SWE-11-NRYAOUTMP**	**Effluent**	**0.6 l filtered**	**22.11.21**	**Gothenburg Ryaverket WWTP**	**57.6972, 11.8901**	**129**
	**SWE-12-NRYAOUT**	**Effluent**	**1.55 l filtered**	**22.11.21**	**Gothenburg Ryaverket WWTP**	**57.6972, 11.8901**	**126**
	**SWE-13-NRYAIN**	**Influent**	**500 ml filtered**	**29.11.21**	**Gothenburg Ryaverket WWTP**	**57.6972, 11.8901**	**406**
	SWE-14-NFINN	Freshwater	4.8 l filtered	22.11.21	Finnsjön, drinking water lake	57.6339, 12.1492	33,6
	**SWE-15-NSOIL1**	**Soil**	**250 mg**	**22.11.21**	**Greggered pasture**	**57.6085, 12.1529**	**131**
	**SWE-16-NSOIL2**	**Soil**	**250 mg**	**22.11.21**	**Greggered pasture**	**57.6085, 12.1529**	**202**
	**SWE-17-NSOIL3**	**Soil**	**250 mg**	**22.11.21**	**Greggered pasture**	**57.6085, 12.1529**	**195**
France	FRA-1-SEINE	Freshwater	2 l filtered	07.12.20	Seine river	48.8842, 2.1642	8
	FRA-2-HOSPWW	Wastewater	0.250 l filtered	16.12.20	Paris Bichat Hospital	48.8981, 2.3323	8
Germany	**GER-1-KREISCHAIN**	**Influent**	**0.050 l filtered**	**28.07.21**	**Kreischa influent**	**50.9622, 13.6387**	**98**
	**GER-2-GROSSESOIL**	**Soil**	**250 mg**	**21.06.21**	**Grosse Garten Park**	**51.0332, 13.7626**	**76**
	**GER-3-ELBEWATER**	**Freshwater**	**1.5 l filtered**	**15.07.21**	**Elbe river**	**51.1117, 13.5724**	**60**
	**GER-4-KLINGSOIL**	**Soil**	**250 mg**	**05.05.21**	**Klingenberg area**	**50.9066, 13.5347**	**57.4**
	**GER-5-KREISCHAOUT**	**Effluent**	**100 ml**	**28.07.21**	**Kreischa effluent**	**50.9622, 13.6387**	**54**
	**GER-6-LWBWATER**	**Freshwater**	**1.5 l filtered**	**04.08.21**	**Lockwitzbach stream**	**50.9373, 13.7733**	**45.5**
	GER-7-LWBSED	Sediment	250 mg	04.08.21	Lockwitzbach stream	50.9373, 13.7733	21.8
	**GER-8-LAKEWATER**	**Freshwater**	**1.5 l filtered**	**26.07.21**	**Leupen Bathing Lake**	**50.9373, 13.7733**	**39.6**
	**GER-9-ELBESED**	**Sediment**	**250 mg**	**15.07.21**	**Elbe RIver**	**51.1117, 13.5724**	**33.4**
	GER-10-GROSSEWATER	Freshwater	1.5 l filtered	21.06.21	Local fountain in Grosse Garten	51.0332, 13.7626	11
	GER-11-KLINGDRINK	Freshwater	1.5 l filtered	05.05.21	Klingenberg drinking water lake	50.9066, 13.5347	9.1
	GER-12-WANNDRINK	Freshwater	1.5 l filtered	19.07.21	Wahnbach drinking water lake (Treated water)	50.8800, 7.3445	12
	GER-13-KLINGSED	Sediment	250 mg	05.05.21	Klingenberg drinking water lake	50.9066, 13.5347	10.1
	GER-14-ELBEFISH	Fish mucus	cotton swab in 250 μl + filtering	15.07.21	Elbe River	51.1117, 13.5724	4.46
	GER-15-LAKESED	Sediment	250 mg	26.07.21	Leupen Bathing Lake	51.0156, 13.8233	2.94
	GER-16-WANNWATER	Freshwater	1.5 l filtered	19.07.21	Wahnbach drinking water lake (Pre-treated water)	50.8800, 7.3445	2.04
Pakistan	**PAK-1-SHAHZAD**	**topsoil**	**250 mg**	**06.02.2021**	**Shahzad Wheat Farm**	**33.6608, 73.1449**	**42.3**
	**PAK-2-NARC**	**topsoil**	**250 mg**	**06.02.2021**	**NARC Chilli farm**	**33.4037, 73.0809**	**38.2**
	PAK-3-QAU	topsoil	250 mg	06.08.2021	QAU botanical garden	33.7367, 73.1607	9.5
	PAK-4-BANI	topsoil	250 mg	06.08.2021	Pasture Bani Gala	33.4304, 73.0910	13.7
	**PAK-5-LAKE**	**Freshwater**	**1 l filtered**	**15.06.2021**	**Lake view stream**	**33.7025, 73.1261**	**23.4**
	PAK-6-SHAHDARA	Freshwater	1 l filtered	15.06.2021	Shahdara stream	33.7025, 73.1261	9.4
	PAK-7-JINNAH	Freshwater	1 l filtered	16.06.2021	Jinnah stream	33.7442, 73.1163	10.1
	PAK-8-BARI	Freshwater	1 l filtered	16.06.2021	Bari Imam stream	33.7442, 73.1163	18.5
	PAK-9-CDAIN	Influent	1 l filtered	22.06.2021	Capital Development Authority, Sewage Treatment Plant	33.3240, 73.0748	19.6
	**PAK-10-CDAOUT**	**Effluent**	**1 l filtered**	**22.06.2021**	**Capital Development Authority, Sewage Treatment Plant**	**33.3240, 73.0748**	**27.9**
	PAK-11-QUAIN	Influent	1 l filtered	23.06.2021	QAU,WWTP	33.4436, 73.0749	8.27
	PAK-12-QUAOUT	Effluent	1 l filtered	23.06.2021	QAU,WWTP	33.4436, 73.0749	12.3

WWTP: wastewater treatment plant; NARC: national agriculture research center; QAU: Quaid-i-Azam University; bold: sample analyzed using functional metagenomics.

### Characterization of clones associated with an increased cefiderocol MIC

**Figure 2:**
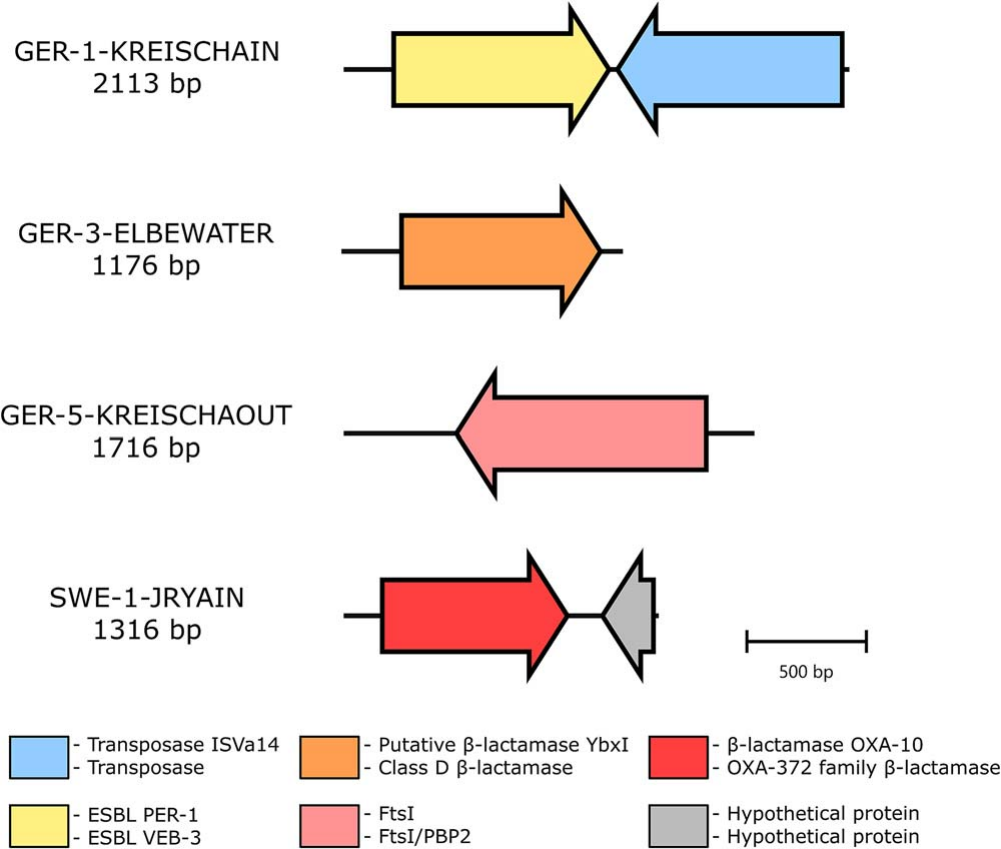
Genetic cartography of insert sequences containing a cefiderocol resistance gene identified using functional metagenomics. ORFs and annotations were obtained using PROKKA v1.14 (top) and Bakta web server v1.11.0 (bottom). The cartography was made using clinker v0.0.21. bp: base pair.

Three of the four identified clones expressed ꞵ-lactamases. Two encoded class D ꞵ-lactamases: an OXA-like ꞵ-lactamase originating from the SWE-1-JRYAIN influent wastewater sample, and a YbxI-like ꞵ-lactamase originating from the GER-3-ELBEWATER Elbe River sample. These clones exhibited cefiderocol MICs of 2 mg/l and 4 mg/l respectively (Table 2). Both were resistant to penicillins (with or without ꞵ-lactamase inhibitors, such as clavulanic acid or tazobactam), to temocillin, and to ceftazidime. Both also showed a synergistic effect between cefoxitin and ceftazidime, and between cefoxitin and cefuroxime (Table 3, Fig. S3A and B). Specifically, the OXA-like encoding clone was also resistant to cefuroxime, cefepime, and to combinations: ceftazidime-avibactam and ceftolozane-tazobactam. The YbxI-like expressing clone showed an additional synergistic effect between cefepime and amoxicillin-clavulanic acid. The *bla*_OXA_-like gene was found on a 1316 bp insert that mapped in the nt database to a *Citrobacter freundii* sequence (91.4% identity and 71% coverage). It was annotated as bearing a hypothetical protein and an OXA-10 or OXA-372 class D ꞵ-lactamase encoding genes (Table 2, Fig. 2). The *bla*_OXA-372_ gene was also the best match in the ResFinder 4.6.0 database (93.2% identity and 100% coverage). The encoded protein matched perfectly in the nr database with a class D ꞵ-lactamase from *Pseudomonas aeruginosa*. Phylogenetically, the OXA-like ꞵ-lactamase was confirmed to be closely related to variants such as OXA-372 (as predicted using ResFinder 4.6.0), OXA-641, and OXA-1016 (Fig. S4A). It was also distant from OXA-427, a known cefiderocol resistance associated variant. The *ybxI*-like gene was found on a 1176 bp insert (Table 2, Fig. 2). In the NCBI nt database, the insert closest homologs were a sequence from bacterium BFN5 (76.6% identity, 57% coverage) and bacteria from the *Bacillota* phylum. Annotation tools identified a single ORF annotated as a class D ꞵ-lactamase or a putative ꞵ-lactamase YbxI encoding gene. This *ybxI*-like gene was not identified in any ARG databases. Only homologous amino acid sequences were found encoded by *Bacillota* bacteria with 68.9 to 78.0% identity in the nr database. Among class D ꞵ-lactamases, the closest variant was an YbxI ꞵ-lactamase (Fig. S4A).

**Table 2:** Molecular characteristics of insert sequences and associated ARGs responsible for a cefiderocol MIC increase.

Sample	Origin	Insert Size bp	FDC MIC mg/l	Insert taxonomy (BLASTN, nt)				Insert annotation		Putative ARG variant in ARG database						Putative ARG encoded protein (BLASTP, nr)				
				Scientific name	id (%)	qcov (%)	Accession number	PROKKA (v1.14.6)	BAKTA (v1.11.0)	ResFinder 4 (v4.6.0)			ResFinderFG (v2.0)			Description	Scientific name	id (%)	qcov (%)	Accession number
										Gene	id (%)	qcov (%)	Gene	id (%)	qcov (%)					
SWE-1- JRAYIN	Waste -water influent	1316	2	*C. freundii*	91.4	71	KP851978.1	- β-lactamase OXA-10 - Hypothetical protein	- OXA-372 family carbapenem-hydrolyzing β-lactamase - Hypothetical protein	*bla*_OXA-372_	93.2	100	-	-	-	class D β-lactamase	*P.* *aeruginosa*	100	100	ELM3777047.1
				*Morganella morganii*	97.2	59	MH211331.1									class D β-lactamase	*P.* *aeruginosa*	99.6	100	ELP2778955.1
				*M. morganii*	97.2	59	NG_057486.1									OXA-641	*M.* *morganii*	96.9	99	WP_109545072.1
				*P. indoloxydans*	96.9	59	NG_076676.1									OXA-1016	*Ectopseudomonas oleovorans*	96.1	99	WP_219860728.1
GER-1-KREISCHAIN	Waste -water influent	2113	1	*A. caviae*	100.0	65	AP022110.1	- ESBL PER-1 - IS4 Transposase ISVa14	- ESBL VEB-3 - Transposase	*bla*_VEB-3_	100.0	100	β-lactamase MG586042.1 Antibiotics polluted stream sediment AMP	99.9	100	ESBL VEB-3	*Gammaproteo-* *bacteria*	100.0	100	WP_020956917.1
				*A. hydrophila*	100.0	54	LC570768.1									ESBL VEB-33	*A.* *veronii*	99.7	100	WP_328703082.1
				*A. pittii*	100.0	54	GQ926879.1									ESBL VEB-9	*Pseudomonadota*	99.7	100	WP_032494864.1
				*K. michiganensis*	100.0	54	CP084543.1									VEB ESBL	*Kiritimatiellia* *bacterium*	99.7	100	MCB1070360.1
GER-3- ELBEWATER	Fresh -water	1176	4	*bacterium* BFN5	76.6	57	CP053389.1	- Putative β-lactamase YbxI	- Class D β-lactamase	-	-	-	-	-	-	putative β-lactamase YbxI	*Sporomusaceae* *bacterium* FL31	75.0	98	GBG55354.1
				*Pelosinus fermentans*	72.6	59	CP010978.1									class D β-lactamase	*bacterium* BFN5	72.4	97	QJW46018.1
				*Peribacillus* sp.	79.1	34	CP133763.1									class D β-lactamase	*Pelosinus sp.* IPA-1	78.0	90	WP_285716311.1
				*Peribacillus simplex*	72.7	46	CP017704.1									class D β-lactamase	*P.* *baikalensis*	68.9	98	WP_229535920.1
GER-5-KREISCHAOUT	Waste -water effluent	1716	2	*L. pneumophila*	64.8	61	CP113439.1	- Peptidoglycan D,D-transpeptidase FtsI	- FtsI/ Penicillin binding protein 2	-	-	-	-	-	-	Penicillin-binding protein	*L. taurinensis*	58.1	99	STY25098.1
				*L. pneumophila*	64.8	61	OZ182546.1									M56 family metallopeptidase	*L. taurinensis*	58.1	99	WP_115301049.1
				*L. pneumophila*	64.8	61	LT906452.1									M56 family metallopeptidase	*L. pneumophila*	57.5	99	WP_129820485.1
				*L. pneumophila*	64.8	61	FQ958210.1									Cell division protein FtsI	*L. pneumophila*	53.6	99	HDO8324588.1

FDC: cefiderocol; bp: base pair; id: identity; qcov: query cover.

**Table 3:** Susceptibility diameters (mm) obtained by disc diffusion assay for clones expressing identified cefiderocol resistance genes.

Strain	Protein expre -ssed	AMO 20 μg	PIL 30 μg	TEM 30 μg	CEF 30 μg	CXM 30 μg	PTZ 30 μg / 6 μg	CTX 5 μg	CLT 30 μg / 10 μg	FOX 30 μg	CZD 10 μg	AMC 20 μg / 10 μg	FEP 30 μg	ETP 10 μg	MEM 10 μg	IPM 10 μg	CZA 10 μg / 4 μg
K12 MG1655 - pHSG199	None	21	28	22	24	26	28	38	29	26	30	24	38	38	34	36	31
SWE-1-JRAYIN	OXA-like	**6**	**9**	**9**	21	**15**	**11**	20	**6**	27	**6**	**10**	**20**	27	28	26	**10**
GER-1-KREISCHAIN	VEB-3	**6**	**14**	22	**6**	**6**	28	**16**	22	27	**6**	23	**18**	34	34	34	16
GER-3-ELBE WATER	YbxI-like	**6**	**8**	**8**	23	24	**17**	38	23	24	**7**	**13**	28	29	32	32	18
GER-5-KREISCHAOUT	PBP-like	20	20	18	19	21	22	28	25	21	**14**	20	30	31	30	29	14

The clone associated with the GER-1-KREISCHAIN influent wastewater sample encoded a VEB-3 class A ꞵ-lactamase, which was responsible for an 8-fold cefiderocol MIC increase (1 mg/l; Table 2). The clone displayed an ESBL profile, with synergistic effects observed for inhibitors (clavulanic acid or tazobactam) and cefotaxime, ceftazidime, and cefepime (Table 3, Fig. S3C). Cefoxitin, temocillin, and carbapenems remained effective. The ꞵ-lactamase encoding gene was found on a 2113 bp insert, mapping in nt database with sequences from Gammaproteobacteria (e.g. *Aeromonas* species and *Klebsiella michiganensis*), albeit with a partial coverage (ranging from 54 to 65%; Table 2, Fig. 2). Annotations for extended-spectrum class A ꞵ-lactamase VEB-3 and an IS*4* family transposase were found on the insert, potentially explaining the previously observed partial coverage. The *bla*_VEB-3_ gene identification was confirmed as it was found in ResFinder 4.6.0. The encoded ꞵ-lactamase was found in the nr database as in Gammaproteobacteria, including Gram-negative pathogens from the ESKAPEE list (e.g. *Enterobacter cloacae*, *E. coli*, *Klebsiella pneumoniae*, *and P. aeruginosa*). Its structure was compared with that of VEB-1 (Accession number: ABD49192.1), a variant for which cefiderocol hydrolysis was previously tested and resulted negative. Despite two amino acid substitutions, the structures align closely, indicating that these differences do not significantly alter the tertiary structure (Fig. S5). Among class A ꞵ-lactamases, the closest ꞵ-lactamases were PER ꞵ-lactamases, three of four of which are associated with increased cefiderocol MICs reaching EUCAST clinical breakpoint (Fig. S4B). Within the VEB family, the VEB-3 ꞵ-lactamase was well-integrated and does not represent a divergent lineage. It was closely related to VEB-32 and VEB-33 (Fig. S4C).

**Figure 3:**
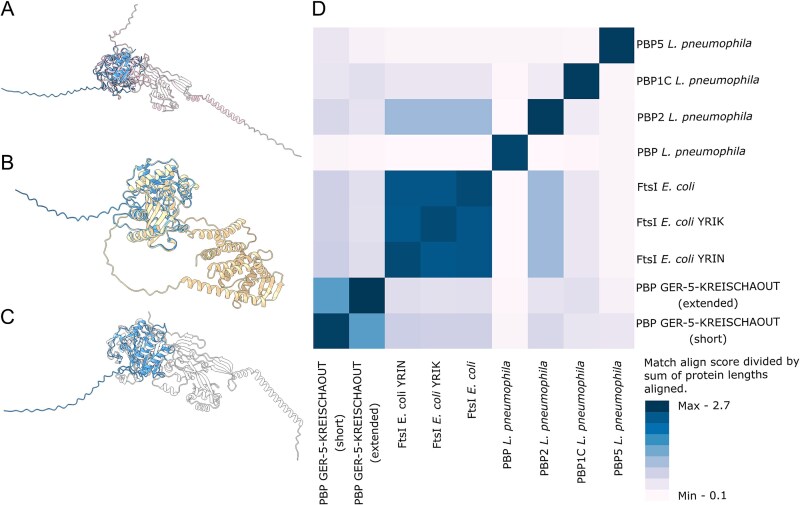
Structures predicted by AlphaFold 3 and aligned using PyMOL. A. FtsI from GER-5-KREISCHAOUT (short) *vs E. coli* FtsI; B. FtsI from GER-5-KREISCHAOUT (short) *vs* FtsI from GER-5-KREISCHAOUT (full-length); C. FtsI from GER-5-KREISCHAOUT (short) *vs L. pneumophila* PBP-2; D. Heatmap showing match align score divided by sum of both protein lengths aligned.

A clone expressing a truncated PBP was identified using the functional metagenomic DNA library from the GER-5-KREISCHAOUT effluent sample, which was collected downstream of the GER-1-KREISCHAIN sample wastewater treatment plant. This clone exhibited a 16-fold cefiderocol-MIC increase (2 mg/l; Table 2) and an atypical profile: low-level resistance only to ceftazidime, with synergistic effect observed between ceftazidime and clavulanic acid or cefoxitin (Table 3, Fig. S3D). Its insert sequence, 1716 bp in length, exhibited 64.8% identity (61% coverage) with a *Legionella pneumophila* sequence (Table 2, Fig. 2). Annotation tools identified a single ORF as a cell division protein FtsI/PBP-2 encoding gene. No homologous sequence was found in tested ARG databases and the closest protein match in the nr database was a PBP found in *Legionella taurinensis* (58.1% identity and 99% coverage). A nitrocefin hydrolysis test was negative and cefiderocol MICs did not differ with or without avibactam (the absence of inhibition activity of avibactam alone was confirmed). Its structure prediction revealed a domain that could be aligned to a specific domain of *E. coli* FtsI and *L. pneumophila* PBP-2 (Fig. 3A and C). However, this also indicated that the protein lacked a substantial portion. This was confirmed, as the *pbp*-like gene was found on a 23 400 bp metagenomic contig from GER-5-KREISCHAOUT metagenomic assembly, with 882 additional base pairs due to the presence of an upstream start codon (Fig. S6). No phenotypic difference was observed between the short and full-length gene-encoding clones. Protein structure alignments revealed the highest similarity between the short and full-length version of the protein identified through functional metagenomics (Fig. 3D). The second-best alignment was with *E. coli* FtsI, followed by *E. coli* FtsI with YRIN/YRIK insertions, and then by *L. pneumophila* PBP-2.

### Cefiderocol ARGs distribution

**Figure 4:**
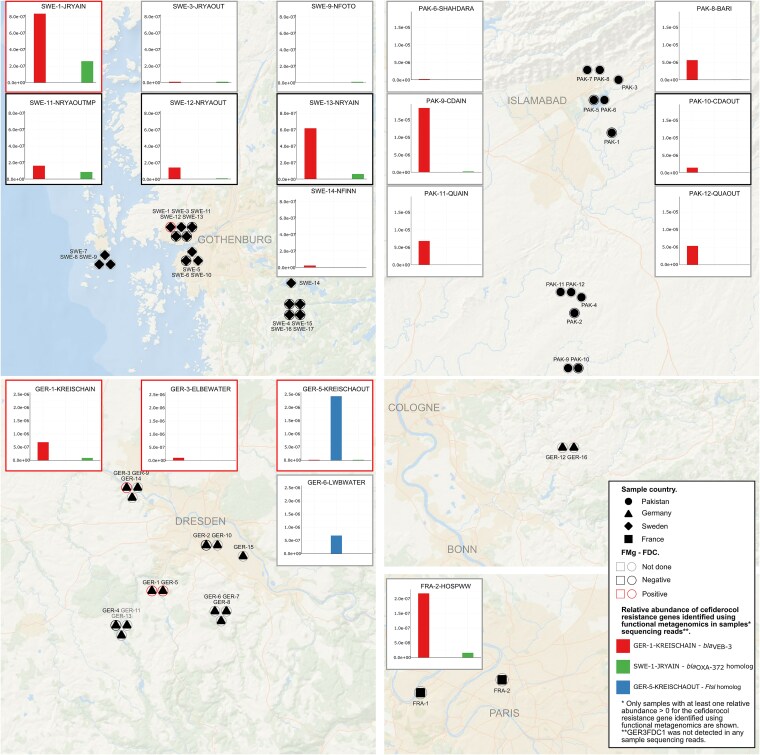
Geographic distribution and relative abundance of cefiderocol resistance genes. Relative abundances of the identified cefiderocol resistance genes in whole metagenome reads are shown if at least one gene was detected (relative abundance >0).

The *bla*_VEB-3_ gene from GER-1-KREISCHAIN was detected in all countries and was the most prevalent of the four functionally identified cefiderocol resistance genes (16/47 positive samples, Table S3), with a relative abundance up to 1.5E-5 (mapped reads/total reads) in samples from Pakistan (Fig. 4). The oxacillinase-encoding gene from SWE-1-JRYAIN was detected in every country but at lower abundances. These two genes exhibited a broad distribution in wastewater and freshwater samples (18/36). All wastewater samples were positive. The *pbp*-like gene found in the GER-5-KREISCHAOUT wastewater sample was present in the freshwater sample GER-6-LWBWATER, which is also located in the Dresden region of Germany. The cefiderocol-resistance gene identified in GER-3-ELBEWATER was not detected in any sample metagenomic reads. None of the genes identified through functional metagenomics were found in soil sample sequencing reads. Homologous proteins of the PBP-like, YbxI-like, and OXA-like proteins in the GMGC catalogue showed only limited sequence identity (ranging from 53.9% to 68.9%). The closest homolog to the VEB-3 ꞵ-lactamase was the GMGC10.001_990_215.UNKNOWN unigene from *P. aeruginosa* (99.3% identity). This unigene was found in 2/7059 human gut samples, 2/1139 human skin samples, and 3/22 wastewater samples. Regarding the host distribution in EnteroBase, only homologs of *bla*_VEB-3_ from GER-1-KREISCHAIN were identified in nine *E. coli* samples (97.8%–97.9% identity and 100% coverage; Table S4). These *E. coli* isolates belonged to phylogroups C (three isolates) and A (six isolates), and included sequence types ST176 (four isolates), ST88 (one isolate), ST472 (one isolate), ST10 (one isolate), ST471 (one isolate), and ST410 (one isolate).

## Discussion

Functional metagenomics identified four distinct cefiderocol resistance genes. Distribution analysis demonstrated ubiquitous detection in all analyzed wastewater samples and presence in the genomes of pathogenic bacteria.

Two class D ꞵ-lactamase encoding gene variants were identified: a *ybxI* and a *bla*_OXA-372_ gene variants. YbxI, previously described in *Bacillus subtilis* as a low activity ꞵ-lactamase, was not previously associated with cefiderocol resistance [50]. However, the YbxI-like ꞵ-lactamase conferred the highest cefiderocol MIC observed in this study above the EUCAST clinical breakpoint. Previously, oxacillinases were not associated with cefiderocol MIC increase [10–12], except OXA-427, which has been associated with cefiderocol resistance in clinical isolates and when expressed by *P. aeruginosa* [51]. Additionally, an environmental *E. cloacae* complex strain encoding OXA-181 (potentially among other resistance mechanisms) was found associated with increased cefiderocol MIC [52]. The OXA-372-like ꞵ-lactamase identified in this study is the second OXA ꞵ-lactamase to be identified as responsible for an increase in cefiderocol MIC, reaching the EUCAST clinical breakpoint (2 mg/l).

Class A ꞵ-lactamases (e.g. PER, SHV, GES, KPC, and BEL) have been associated with elevated MICs upon cloning into *E. coli* or *P. aeruginosa* (from 2-fold to 67-fold). The SHV-12, PER-1, PER-6, and PER-7 ꞵ-lactamases conferred MIC exceeding the EUCAST clinical breakpoint. In contrast, the VEB ꞵ-lactamase family was not previously associated with increased cefiderocol MICs or its hydrolysis [8, 12]. VEB-1 was previously shown to be unable to hydrolyze cefiderocol. Although *P. aeruginosa* clinical isolates producing VEB ꞵ-lactamases have been reported with cefiderocol MICs >1 mg/l, additional resistance mechanisms may have contributed to the observed MIC increase [9]. In this study, the VEB-3 ꞵ-lactamase identified by functional metagenomics was responsible for a cefiderocol MIC increase (1 mg/l). This value remains below EUCAST clinical breakpoint. However, in *Enterobacterales* isolates, the association of ESBL production and porin loss was the second most frequent resistance mechanism observed in a collection of isolates with cefiderocol MIC >2 mg/l [6]. Therefore, as with other ESBLs associated with other resistance mechanisms, VEB-3 expression could contribute to a cefiderocol resistance phenotype.

Functional metagenomics also identified a partial *pbp*-like gene responsible for a 16-fold increase in cefiderocol MIC, which reached the EUCAST clinical breakpoint. The full-length protein showed an identical phenotypic profile, and its sequence homology suggested it originates from the genus *Legionella*. Existing data lack information on the in vitro or in vivo activity of cefiderocol against this genus, which is composed of species acting as intracellular pathogens. Previous studies showed that cloning and expressing the *E. coli* PBP-3 encoding gene alone was not associated with elevated cefiderocol MIC. Modifications of the PBP-3 target, specifically YRIN or YRIK motif insertions at position 338, have been linked to cefiderocol MIC increase. The former has been associated with a 2-fold cefiderocol MIC increase but, in contrast to the partial *pbp*-like gene found in this study, did not reach EUCAST clinical breakpoint [8, 13, 21, 22].

The mechanistic basis of cefiderocol resistance is complex, often involving multiple ARGs, virulence factors, and/or mutations. Functional metagenomics is a qualitative technique that eliminates the need for prior assumptions, allowing for the cloning of DNA fragments and the direct selection of genes of interest. This approach is less laborious than the cloning of individual ARGs into a susceptible host (which often results in negative outcomes) and accelerates the identification of new phenotypic information regarding known ARGs and the discovery of previously uncharacterized resistance genes. However, it possesses inherent limitations: expression biases, insert size limitations, and expression of DNA fragments that might be toxic for the host cell. Another important setting is the antibiotic concentration used for selection. We chose a 1 mg/l cefiderocol concentration for selection. It ensures robust selection of genes contributing to increasing cefiderocol MIC and helps mitigate technical issues associated with working at low antibiotic concentrations. We acknowledge that this threshold might have missed mechanisms responsible for cefiderocol MIC increase <1 mg/l. Furthermore, as a qualitative technique, it does not provide an exhaustive description of all ARGs within a sample. This is exemplified in this study, where resistance genes identified through functional metagenomics were not consistently detected in corresponding metagenomic sequence data, and genes detected in metagenomic sequence data were not always identified by functional metagenomics.

Once previously uncharacterized ARGs are described, it is essential to assess their associated dissemination potential. One of the functionally identified ARG was detected in 18 out of 47 environmental metagenomes analyzed, with 100% of wastewater metagenomes testing positive for this gene. Wastewater, known to harbor diverse microbial communities including pathogenic bacteria and their genetic content (ARGs, virulence factors, mobile genetic elements), has been identified as a hotspot for ARG mobilization and promotion [33, 53]. In addition to the potential for monitoring population-wide resistance, this has persuaded authorities (e.g. in the European Union and Australia) to pursue systematic antimicrobial surveillance in urban wastewater treatment plans [54, 55]. The *pbp*-like gene exhibited a narrow distribution, likely due to the usual chromosomal location of *pbp* genes and the absence of associated mobility. Conversely, *bla*_VEB-3_ displayed a broad distribution, which is probably a result of its association with a mobile genetic element. In contrast, soil is often considered a potential reservoir for resistance [35]. However, the ARGs identified by functional metagenomics were not detected in soil metagenomes. ARGs identified using functional metagenomics had homologs or were found in bacterial isolates from the ESKAPEE list [56–61]. Homologs were identified in genomes from the Enterobase database: in ST410 *E. coli* strains, a disseminating ST, which is considered a high-risk multi-drug resistant clone causing human disease [62, 63]. These results demonstrate the value of the qualitative description using functional metagenomics. Identifying these previously uncharacterized ARGs and acquiring new phenotypic data significantly enhance the accuracy of resistome description in the environment and in clinical isolate genomes.

## Supplementary Material

ISME_Supp_wo_modif_wrag010

## Data Availability

Scripts and software versions used throughout this study are available on the github repository: https://github.com/RemiGSC/Mgf_FDC/ and on the following Zenodo doi:https://doi.org/10.5281/zenodo.15487633. The raw metagenomic and genomic sequencing data generated and analyzed in this study have been deposited in the NCBI Sequence Read Archive (SRA) under the BioProject accession number PRJNA1262354. Accession numbers for whole genome sequencing of cefiderocol resistance clones are: SAMN48516469, SAMN48516470, SAMN48516471, SAMN48516472. The accession number of WGS of the control K12 *E. coli* host transformed with empty pHSG299 is: SAMN48516473.
