# Supplementary material for: Cefiderocol resistance genes identified in environmental samples using functional metagenomics
Source: ISME J. 2026 Jan 28;20(1):wrag010. doi: 10.1093/ismejo/wrag010 (PMC12915585; doi:10.1093/ismejo/wrag010)
Supplement: ISME_Supp_wo_modif_wrag010 [file isme_supp_wo_modif_wrag010.docx]

**Cefiderocol resistance genes identified in environmental samples using functional metagenomics.**

**Supplementary materials and methods**

DNA isolation

Water and wastewater samples were filtered through 0.22 µm MF-Millipore filters (Merck, Darmstadt, Germany) prior to extraction. Filtration volumes varied: 1-6 L for freshwater or saltwater samples, and 0.05-1 L for wastewater input or output. The filters were then shredded and processed according to the kit extraction protocol. In total, 250 mg of material was used per extraction tube for the soil samples. Regarding the fish sample, a European bullhead (*Cottus gobio*) was removed from water of the Elbe River in Dresden, Germany, and its skin mucus samples taken immediately after the fish was removed from the water using sterile cotton swabs [[1]](https://www.zotero.org/google-docs/?REZonf). The collected mucus swab was transferred into a PowerBead tube and stored at −20°C before DNA extraction. Fishing and animal handling were carried out in accordance with federal legislation and ethics approval based on permits issued by the Saxon State Office for Environment, Agriculture, and Geology (AZ 76/1/9222.22–03/22). Ethical aspects of sampling were conducted following the requirements of Directive 2010/63/EU of the European Parliament and of the Council of 22 September 2010 on the protection of animals used for scientific purposes.

Functional metagenomic libraries

DNA was sheared using the tagmentase enzyme from the Nextera XT kit (Illumina, San Diego, USA) following an optimized protocol. To achieve a target size of 1-3 kb, DNA was incubated with a 1/10 dilution of the tagmentase enzyme for 10 seconds at 55°C. The reaction was quickly transferred on ice, and the sheared DNA was purified using QIAquick PCR Purification Kit (Qiagen, Hilden, Germany). The tagmented DNA was amplified using primers designed for adapter ligation, incorporating overlaps necessary for Gibson assembly. DNA fragments within the 1-3 kb range were size selected and purified using QIAquick Gel Extraction Kit (Qiagen, Hilden, Germany).

The expression vector used was pHSG299 (Takara Bio, Shiga, Japan; accession number: M19415). The vector was linearized by PCR using primers designed for adapter ligation, incorporating complementary overlaps required for Gibson cloning.

Gibson assembly was performed using NEBuilder HiFi DNA Assembly (New England Biolabs, Ipswich, USA). The Gibson assembly reaction mixture containing 0.1 pmol inserts and 0.05 pmol vector was dialyzed against a 0.025 µm MF-Millipore membrane (Merck, Darmstadt, Germany). *Escherichia coli* K12 MG1655 cells were made competent by multiple centrifugation and washes at 4°C in 10% glycerol. A 15 µL aliquot of the Gibson assembly reaction mixture was combined with 35 µL of competent cells, mixed, and then transferred in a 1 mm electroporation cuvette. Cells were electroporated at 1800V using an Eppendorf electroporator 2510 and subsequently resuspended in 1 mL Luria-Bertani (LB) media. The cell suspension was incubated for 2 hours at 37°C.

Cells were centrifuged (8000 rpm, 4 min), resuspended in 400 µL of LB medium, and 100 µL was used for serial dilutions and plating on LB agar media containing kanamycin (50 mg/L) incubated overnight at 37°C. To ensure cloning efficiency of 1 to 3 kb inserts, 15 clones were randomly selected and subjected to PCR amplifying the insertion site. Amplicons were pooled, and the pool size was analyzed using a Tape Station (Agilent, USA). The remaining cells were used to inoculate 50 mL LB broth containing 50 mg/L kanamycin for overnight incubation at 30°C. Cells were then centrifuged (8000 rpm, 4 min) and resuspended in 8 mL LB+Glycerol (15%) for storage at -80°C.

Clone selection

To select for cefiderocol-resistant clones from the functional metagenomic libraries, *E. coli* K12 MG1655 carrying libraries were plated on LB agar media supplemented with 100 mM IPTG and 1 mg/L cefiderocol to select for significant MIC increase and to avoid potential false positives associated with the use of low antibiotic concentrations. The use of iron-depleted media was not required as iron is sufficiently bound to agar, mimicking an iron-depleted state [[2]](https://www.zotero.org/google-docs/?HeKaKe). To ensure the resistance phenotype was attributable to DNA cloned in the expression vector, the cefiderocol-resistant colonies were selected as follows: 1) Clones were resuspended in a 20 µL NaCl solution. 2) The bacterial suspension was used to inoculate two separate LB agar plates, one supplemented with kanamycin (50 mg/L) and the other with 100 mM IPTG and 1 mg/L cefiderocol. 3) The remaining bacterial suspension was heated at 95°C and subjected to PCR amplification, targeting the vector insertion site to amplify the inserted DNA fragment. Clones exhibiting growth on both selective media and yielding a positive PCR result were stored at -80°C in LB medium containing 15% glycerol.

Confirmation of plasmid mediated cefiderocol resistance

To further confirm plasmid-mediated cefiderocol resistance, plasmids presumably containing a cefiderocol resistance gene were extracted and purified from each resistant clone using the Plasmid Mini Kit (Qiagen, Hilden, Germany). Each purified plasmid was then used to transform fresh competent *E. coli* K12 cells. The same electroporation protocol was performed, diluting 15 µL of plasmid containing insert (0.05 pmol) in 35 µL of competent *E. coli* cells. A phenotypic characterization of the transformants was performed and compared to the initial resistant clone phenotype.

Additionally, each resistant clone underwent whole genome sequencing (WGS) to discard potential mutations associated with increased cefiderocol MIC. Genomic DNA was extracted using EZ1&2, DNA Tissue kit (Qiagen, Hilden, Germany). Libraries for WGS were prepared using Nextera DNA Flex kit and sequenced on a NextSeq (Illumina, San Diego, USA). Reads were quality filtered, and the clone genome was assembled using SPAdes v3.11.1 [[3]](https://www.zotero.org/google-docs/?dd9MiU). Assembly was annotated using PROKKA v1.14, and potential mutations in genes previously associated with cefiderocol resistance (**Table S2**) were investigated by comparing their sequence to genes found in the *E. coli* K12 MG1655 transformed with empty pHSG299. Whole genome sequencing data was also analyzed to find potential mutations using Parsnp with default parameters [[4]](https://www.zotero.org/google-docs/?kqCffK).

Phenotypic characterization

Overnight culture was performed using LB agar media containing 100 mM IPTG and 1 mg/L cefiderocol. Several colonies were picked to inoculate a 0.9% NaCl solution to obtain a 0.5 McFarland suspension used for MIC testing and disc diffusion assay, following the European Committee on Antimicrobial Susceptibility Testing (EUCAST) recommendations. MICs were determined in triplicates using unitary UMIC Cefiderocol tests (Bruker, Billerica, USA) in iron-depleted cation-adjusted Mueller Hinton (CAMH) broth (Biocentric, France). Disc diffusion assays were also performed to assess susceptibility to a range of ꞵ-lactam antibiotics and ꞵ-lactam-ꞵ-lactamase inhibitor combinations. To further investigate ꞵ-lactamase mediated resistance or the potential effect of the ꞵ-lactamase inhibitor, a nitrocefin-hydrolysis test was performed along with avibactam and cefiderocol-avibactam (4 mg/L) MICs determination in triplicate using unitary UMIC Cefiderocol tests (Bruker, Billerica, USA) in iron-depleted CAMH broth. Additionally, to assess the intrinsic effect of avibactam alone, avibactam MICs were determined in triplicate in CAMH broth with avibactam concentrations ranging from 0 to 256 mg/L.

Phylogenetic analysis

In addition to the sequences of the described genes, other ARG sequences were collected from cloning publications with available data on the single-gene cefiderocol MIC [[5]](https://www.zotero.org/google-docs/?xgeUlX). The alignments were performed by the EMBL-EBI web version of Clustal Omega. Phylogenetic trees were built using IQ-TREE v2.2.0, with ModelFinder. The best amino acid substitution models were thus retained: Q.pfam+G4 for Ambler class A and class D ꞵ-lactamases, and Q.yeast+G4 for the VEB family. These two models use amino acid exchange matrices, trained respectively on the PFAM database (Q.pfam) and on yeast protein data (Q.yeast), with the addition in both models of a discrete gamma distribution with 4 categories (G4). Of note, no amino acid substitution matrix was trained specifically on proteins from prokaryotes by the authors of QMaker; they are thus absent from ModelFinder [[6]](https://www.zotero.org/google-docs/?PN5miL).

**Supplementary results**

Confirmation of plasmid mediated cefiderocol resistance

Fresh *E. coli* K12 MG1655 transformed with the plasmid containing the identified ARG exhibited either cefiderocol MICs that were equivalent or up to 2-fold higher than those of the original cefiderocol resistant clone. Antibiotic disc diffusion assays also confirmed that both clones displayed the same resistance phenotype. Subsequent genomic DNA sequencing of each resistant clone revealed no mutations in genes previously associated with cefiderocol resistance (**Table S2**). Instead, random mutations were found in specific clones within intergenic regions and in genes encoding rhsC (a transposase), GTPase Der, and a primosomal replication protein N.

**Table S1.** Genes previously associated with cefiderocol resistance.

| Genes associated with cefiderocol resistance | | | | | | | | | | | | |
| --- | --- | --- | --- | --- | --- | --- | --- | --- | --- | --- | --- | --- |
| *abrB* | *baeR* | *baeS* | *bcr* | *ccmB* | *cirA* | *ddlB* | *dppF* | *eamB* | *envZ* | *eutH* | *exbB* | *exbD* |
| *fecA* | *fecB* | *fepA* | *fepB* | *fhuA* | *fiu* | *fruA* | *ftsI* | *gspK* | *ldtA* | *lysP* | *mdtA* | *mdtB* |
| *mdtC* | *mdtI* | *mepS* | *mglA* | *mglB* | *mglC* | *mraY* | *murC* | *murD* | *murE* | *murF* | *murG* | *nupX* |
| *ompC* | *ompK* | *ompL* | *ompR* | *osmF* | *pbpG* | *pcnB* | *phoE* | *proX* | *psuT* | *rcnA* | *secA* | *setB* |
| *shiA* | *tauC* | *tonB* | *tsgA* | *ydcT* | *yebT* | *yedA* | *yeeO* | *yegH* | *yegT* | *yehW* | *yehX* | *yehY* |
| *abrB* | *baeR* | *baeS* | *bcr* | *ccmB* | *cirA* | *ddlB* | *dppF* | *eamB* | *envZ* | *eutH* | *exbB* | *exbD* |
| *fecA* | *fecB* | *fepA* | *fepB* | *fhuA* | *fiu* | *fruA* | *ftsI* | *gspK* | *ldtA* | *lysP* | *mdtA* | *mdtB* |
| *mdtC* | *mdtI* | *mepS* | *mglA* | *mglB* | *mglC* | *mraY* | *murC* | *murD* | *murE* | *murF* | *murG* | *nupX* |
| *ompC* | *ompK* | *ompL* | *ompR* | *osmF* | *pbpG* | *pcnB* | *phoE* | *proX* | *psuT* | *rcnA* | *secA* | *setB* |
| *shiA* | *tauC* | *tonB* | *tsgA* | *ydcT* | *yebT* | *yedA* | *yeeO* | *yegH* | *yegT* | *yehW* | *yehX* | *yehY* |
| *abrB* | *baeR* | *baeS* | *bcr* | *ccmB* | *cirA* | *ddlB* | *dppF* | *eamB* | *envZ* | *eutH* | *exbB* | *exbD* |
| *fecA* | *fecB* | *fepA* | *fepB* | *fhuA* | *fiu* | *fruA* | *ftsI* | *gspK* | *ldtA* | *lysP* | *mdtA* | *mdtB* |
| *mdtC* | *mdtI* | *mepS* | *mglA* | *mglB* | *mglC* | *mraY* | *murC* | *murD* | *murE* | *murF* | *murG* | *nupX* |

**Table S2.** Mutations identified between *E. coli* K12 MG1655 used for functional metagenomics and each clone characterized by an increased cefiderocol MIC.

| #CHROM | POS | ID | REF | ALT | QUAL | FILTER | INFO | FORMAT | K12 | GER3 | GER5 | SWE1 | GER1 | Annotation |
| --- | --- | --- | --- | --- | --- | --- | --- | --- | --- | --- | --- | --- | --- | --- |
| NODE_2_length_297294_cov_64.355229 | 107815 | AAGCTTCGAC.CAAGGTTCGA | C | T | 40 | PASS | NA | GT | 0 | 0 | 1 | 1 | 1 | intergenic |
| NODE_4_length_204794_cov_62.648114 | 115318 | TTTTGCCGCG.TTTACGTACG | T | C | 40 | PASS | NA | GT | 0 | 0 | 0 | 0 | 0 | GTPase Der |
| NODE_9_length_172398_cov_67.719686 | 99060 | GGTGTTGTCC.GGCACCGTGT | G | T | 40 | PASS | NA | GT | 0 | 0 | 0 | 0 | 1 | Primosomal replication protein N |
| NODE_9_length_172398_cov_67.719686 | 99171 | TCACCGGCAG.GCGTGGTGTC | G | C | 40 | PASS | NA | GT | 0 | 0 | 1 | 0 | 0 | Primosomal replication protein N |
| NODE_11_length_147951_cov_66.046628 | 142083 | ACGGCGACCC.ACCAGGCGGC | A | T | 40 | PASS | NA | GT | 0 | 0 | 0 | 1 | 0 | Poly(A) polymerase I |
| NODE_16_length_112849_cov_64.603793 | 384 | CGTCCATCTG.AGCAGCACCG | A | C | 40 | PASS | NA | GT | 0 | 0 | 0 | 1 | 0 | intergenic |
| NODE_16_length_112849_cov_64.603793 | 540 | GGTCGAATGC.ACGAGCAGCA | A | G | 40 | PASS | NA | GT | 0 | 0 | 0 | 1 | 0 | intergenic |
| NODE_17_length_105077_cov_65.529210 | 103816 | TGATAATCAC.TTCGCTCCTT | T | A | 40 | PASS | NA | GT | 0 | 1 | 1 | 1 | 0 | intergenic |
| NODE_17_length_105077_cov_65.529210 | 103819 | TAATCACTTC.GCTCCTTATC | G | T | 40 | PASS | NA | GT | 0 | 1 | 1 | 1 | 0 | intergenic |
| NODE_17_length_105077_cov_65.529210 | 103852 | TTAGTCATGC.TCTAATAAAA | T | C | 40 | PASS | NA | GT | 0 | 1 | 1 | 1 | 0 | intergenic |
| NODE_17_length_105077_cov_65.529210 | 104126 | ATAAAGAAAA.CATCATGGCT | C | A | 40 | PASS | NA | GT | 0 | 1 | 0 | 1 | 0 | putative protein YnaE |
| NODE_17_length_105077_cov_65.529210 | 104532 | ACTATGTCTC.GAATTTTTGC | G | A | 40 | PASS | NA | GT | 0 | 0 | 0 | 1 | 0 | intergenic |
| NODE_17_length_105077_cov_65.529210 | 104827 | TAGCATTGGG.TGGCATTGAC | T | G | 40 | PASS | NA | GT | 0 | 1 | 0 | 1 | 0 | intergenic |
| NODE_17_length_105077_cov_65.529210 | 104971 | TTGGTCGACC.TCCTGTGTTA | T | A | 40 | PASS | NA | GT | 0 | 1 | 1 | 1 | 0 | intergenic |
| NODE_21_length_83857_cov_69.590332 | 45532 | GTGATATACC.TCCCCTGCAG | T | G | 40 | PASS | NA | GT | 0 | 0 | 1 | 0 | 0 | hypothetical protein |
| NODE_21_length_83857_cov_69.590332 | 45803 | TCTGCTCCAC.GGTCAGGCCG | G | C | 40 | PASS | NA | GT | 0 | 1 | 0 | 1 | 0 | hypothetical protein |
| NODE_21_length_83857_cov_69.590332 | 46157 | CCCGTTCACG.TCGCCACACC | T | C | 40 | PASS | NA | GT | 0 | 0 | 1 | 1 | 0 | hypothetical protein |
| NODE_21_length_83857_cov_69.590332 | 46208 | AAAGATAGCG.GCTTTCGACC | G | A | 40 | PASS | NA | GT | 0 | 0 | 1 | 1 | 0 | hypothetical protein |
| NODE_21_length_83857_cov_69.590332 | 49159 | TGCGCGTCAG.ACCGCCCTGC | A | C | 40 | PASS | NA | GT | 0 | 1 | 1 | 1 | 0 | Protein RhsC |
| NODE_21_length_83857_cov_69.590332 | 49176 | TGCGTGGTAC.AGATGGCTTT | A | G | 40 | PASS | NA | GT | 0 | 1 | 1 | 1 | 0 | Protein RhsC |
| NODE_21_length_83857_cov_69.590332 | 49179 | GTGGTACAGA.TGGCTTTTCC | T | C | 40 | PASS | NA | GT | 0 | 1 | 1 | 1 | 0 | Protein RhsC |
| NODE_21_length_83857_cov_69.590332 | 49183 | TACAGATGGC.TTTTCCCCAC | T | C | 40 | PASS | NA | GT | 0 | 1 | 1 | 1 | 0 | Protein RhsC |
| NODE_21_length_83857_cov_69.590332 | 49239 | GCAATGACGG.TGGTCAGGTC | T | C | 40 | PASS | NA | GT | 0 | 1 | 1 | 1 | 0 | Protein RhsC |
| NODE_21_length_83857_cov_69.590332 | 49256 | GTCACCGGCG.GCGTTGTATT | G | A | 40 | PASS | NA | GT | 0 | 1 | 1 | 1 | 0 | Protein RhsC |
| NODE_21_length_83857_cov_69.590332 | 49257 | TCACCGGCGG.CGTTGTATTC | C | T | 40 | PASS | NA | GT | 0 | 1 | 1 | 1 | 0 | Protein RhsC |
| NODE_21_length_83857_cov_69.590332 | 49371 | ACCGCCGTCA.CCTGACCAAA | C | T | 40 | PASS | NA | GT | 0 | 1 | 1 | 1 | 0 | Protein RhsC |
| NODE_21_length_83857_cov_69.590332 | 49375 | CCGTCACCTG.ACCAAAACGG | A | C | 40 | PASS | NA | GT | 0 | 1 | 1 | 1 | 0 | Protein RhsC |
| NODE_21_length_83857_cov_69.590332 | 49378 | TCACCTGACC.AAAACGGTCA | A | G | 40 | PASS | NA | GT | 0 | 1 | 1 | 1 | 0 | Protein RhsC |
| NODE_21_length_83857_cov_69.590332 | 49381 | CCTGACCAAA.ACGGTCATGG | A | G | 40 | PASS | NA | GT | 0 | 1 | 1 | 1 | 0 | Protein RhsC |
| NODE_21_length_83857_cov_69.590332 | 49396 | CATGGTCATA.GCGGGTTACA | G | A | 40 | PASS | NA | GT | 0 | 1 | 1 | 1 | 0 | Protein RhsC |
| NODE_21_length_83857_cov_69.590332 | 49420 | CGGAACAGTC.AGTGAAGCTC | A | G | 40 | PASS | NA | GT | 0 | 1 | 1 | 1 | 0 | Protein RhsC |
| NODE_29_length_56817_cov_65.849595 | 112 | CTGGGCCTTT.TGTTTTATCT | T | C | 40 | LCB | NA | GT | 0 | 1 | 1 | 1 | 0 | intergenic |
| NODE_29_length_56817_cov_65.849595 | 139 | GTCGGTGAAC.ACTCTCCCGA | A | G | 40 | LCB | NA | GT | 0 | 1 | 1 | 1 | 0 | intergenic |
| NODE_29_length_56817_cov_65.849595 | 146 | AACACTCTCC.CGAGTAGGAC | C | T | 40 | LCB | NA | GT | 0 | 1 | 1 | 1 | 0 | intergenic |
| NODE_44_length_21718_cov_67.749318 | 16881 | GGTTGGCCAG.GGTGAATAAC | G | C | 40 | PASS | NA | GT | 0 | 0 | 0 | 0 | 0 | IS5 family transposase ISKpn26 |
| NODE_44_length_21718_cov_67.749318 | 16919 | TTTTTCAGCA.GCCCCTTGTA | G | A | 40 | PASS | NA | GT | 0 | 0 | 0 | 0 | 0 | IS5 family transposase ISKpn26 |
| NODE_44_length_21718_cov_67.749318 | 16953 | AGCCGAACTG.CCGCTTGATG | C | T | 40 | PASS | NA | GT | 0 | 0 | 0 | 0 | 0 | IS5 family transposase ISKpn26 |
| NODE_44_length_21718_cov_67.749318 | 16971 | TGATGCGAAA.CGGGTGCTCC | C | T | 40 | PASS | NA | GT | 0 | 0 | 0 | 0 | 0 | IS5 family transposase ISKpn26 |
| NODE_44_length_21718_cov_67.749318 | 16989 | CCACCCTGGC.ACGGATGCTG | A | C | 40 | PASS | NA | GT | 0 | 0 | 0 | 0 | 0 | IS5 family transposase ISKpn26 |
| NODE_44_length_21718_cov_67.749318 | 17040 | TGTTCTTGCG.CGGATTCTGC | C | T | 40 | PASS | NA | GT | 0 | 0 | 0 | 0 | 0 | IS5 family transposase ISKpn26 |
| NODE_44_length_21718_cov_67.749318 | 17049 | GCGGATTCTG.CTTCAAGGTT | C | T | 40 | PASS | NA | GT | 0 | 0 | 0 | 0 | 0 | IS5 family transposase ISKpn26 |
| NODE_44_length_21718_cov_67.749318 | 17059 | CTTCAAGGTT.TTTACCTTGC | T | C | 40 | PASS | NA | GT | 0 | 0 | 0 | 0 | 0 | IS5 family transposase ISKpn26 |
| NODE_44_length_21718_cov_67.749318 | 17073 | CCTTGCCGGG.ACGCTCGGCG | A | G | 40 | PASS | NA | GT | 0 | 0 | 0 | 0 | 0 | IS5 family transposase ISKpn26 |
| NODE_44_length_21718_cov_67.749318 | 17130 | GCTGTGGCGC.TCCTTGGTAG | T | C | 40 | PASS | NA | GT | 0 | 0 | 1 | 0 | 0 | IS5 family transposase ISKpn26 |
| NODE_44_length_21718_cov_67.749318 | 17385 | CATCGACCAA.AGTGCCTTGG | A | G | 40 | PASS | NA | GT | 0 | 1 | 1 | 0 | 1 | IS5 family transposase ISKpn26 |
| NODE_2_length_297294_cov_64.355229 | 107815 | AAGCTTCGAC.CAAGGTTCGA | C | T | 40 | PASS | NA | GT | 0 | 0 | 1 | 1 | 1 | intergenic |

**Table S3.** Number of samples found to be positive for each gene detection using functional metagenomics (FMg) and/or metagenomics (Mg).

|  | FMg+/Mg+ | FMg-/Mg+ | FMg-/Mg- | FMg+/Mg- | FMgNA/Mg+ | FMgNA/Mg- | Tot. |
| --- | --- | --- | --- | --- | --- | --- | --- |
| *bla*_OXA-like_ | 1 | 6 | 14 | 0 | 7 | 19 | 47 |
| *bla*_VEB-3_ | 1 | 7 | 13 | 0 | 8 | 18 | 47 |
| *pbp*-like | 1 | 1 | 19 | 0 | 0 | 26 | 47 |
| *YbxI* | 0 | 0 | 20 | 1 | 0 | 26 | 47 |

FMg: functional metagenomics, Mg: metagenomics, NA: not done, Tot: total.

**Table S4.** Alignment of the ARGs identified by functional metagenomics using BLASTN against Enterobase database. Only results with pident > 95% and qcovhsp >95% are shown.

| ARG | *E. coli* strain | pident | qcovhsp | mismatch | Gap  open | e  value | bitscore | Serotype | ST | fimH | Phylogroup |
| --- | --- | --- | --- | --- | --- | --- | --- | --- | --- | --- | --- |
| *bla*_VEB-3_ | ESC_KA9999AA_AS | 97,9 | 100 | 19 | 0 | 0.0 | 1569 | O26:H32 | ST10/- | fimH24 | A |
| *bla*_VEB-3_ | ESC_IA4897AA_AS | 97,9 | 100 | 19 | 0 | 0.0 | 1569 | O128:H12 | ST472/- | fimH457 | A |
| *bla*_VEB-3_ | ESC_IA4024AA_AS | 97,9 | 100 | 19 | 0 | 0.0 | 1569 | O129:H30 | ST176/- | fimH23 | A |
| *bla*_VEB-3_ | ESC_NA7870AA_AS | 97,9 | 100 | 19 | 0 | 0.0 | 1569 | Unknown:H4 | ST88/74 | fimH39 | C |
| *bla*_VEB-3_ | ESC_IA4025AA_AS | 97,9 | 100 | 19 | 0 | 0.0 | 1569 | O129:H30 | ST176/- | fimH23 | A |
| *bla*_VEB-3_ | ESC_IA6016AA_AS | 97,9 | 100 | 19 | 0 | 0.0 | 1569 | O129:H30 | ST176/- | fimH23 | A |
| *bla*_VEB-3_ | ESC_LA1083AA_AS | 97,9 | 100 | 19 | 0 | 0.0 | 1569 | O129:H30 | ST176/- | fimH23 | A |
| *bla*_VEB-3_ | ESC_VA2039AA_AS | 97,8 | 100 | 20 | 0 | 0.0 | 1564 | O179:H9 | ST410/471 | fimH24 | C |
| *bla*_VEB-3_ | ESC_RA9125AA_AS | 97,8 | 100 | 20 | 0 | 0.0 | 1564 | O179:H9 | ST-/471 | fimH24 | C |

ARG: antibiotic resistance gene; pident: percentage of identical matches; qcovhsp: query coverage per High-Scoring Pair (HSP); ST: sequence type; fimH: Type 1 fimbriae adhesin.


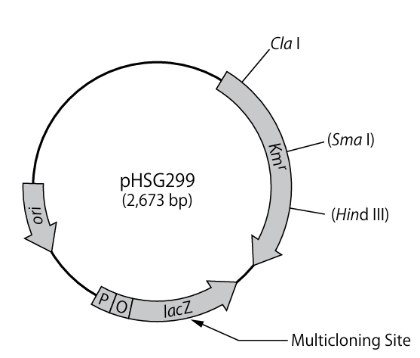


**Figure S1.** Cartography of pHSG299 plasmid (Accession number: M19415; with the following modifications: 1663 C →deletion, 1808 C → T, 2228-2229 AT → TA, 2474 G →A, 26672668 GA→deletion ).


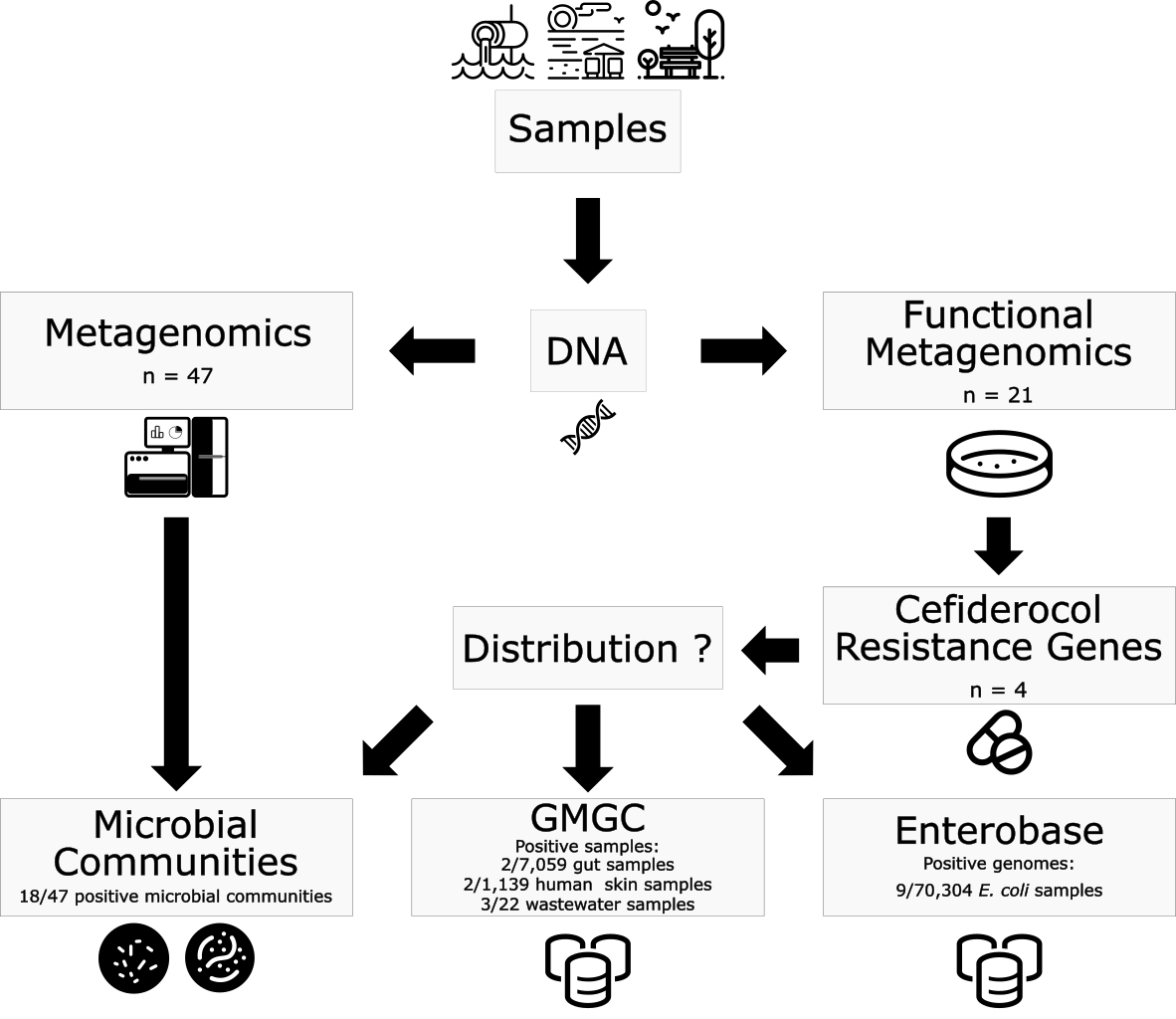


**Figure S2.** Study design and workflow. After DNA isolation, DNA was subjected either to metagenomics alone or to metagenomics and functional metagenomics. The latter was used to identify unknown cefiderocol resistance mechanisms. The distribution and clinical relevance of the newly identified mechanisms were subsequently studied across three levels: 1) in the microbial communities obtained after metagenomic sequencing of each sample; 2) in the global microbial gene catalog; 3) in *E. coli* genomes found in the Enterobase database. FDC: cefiderocol; MIC: minimum inhibitory concentration.


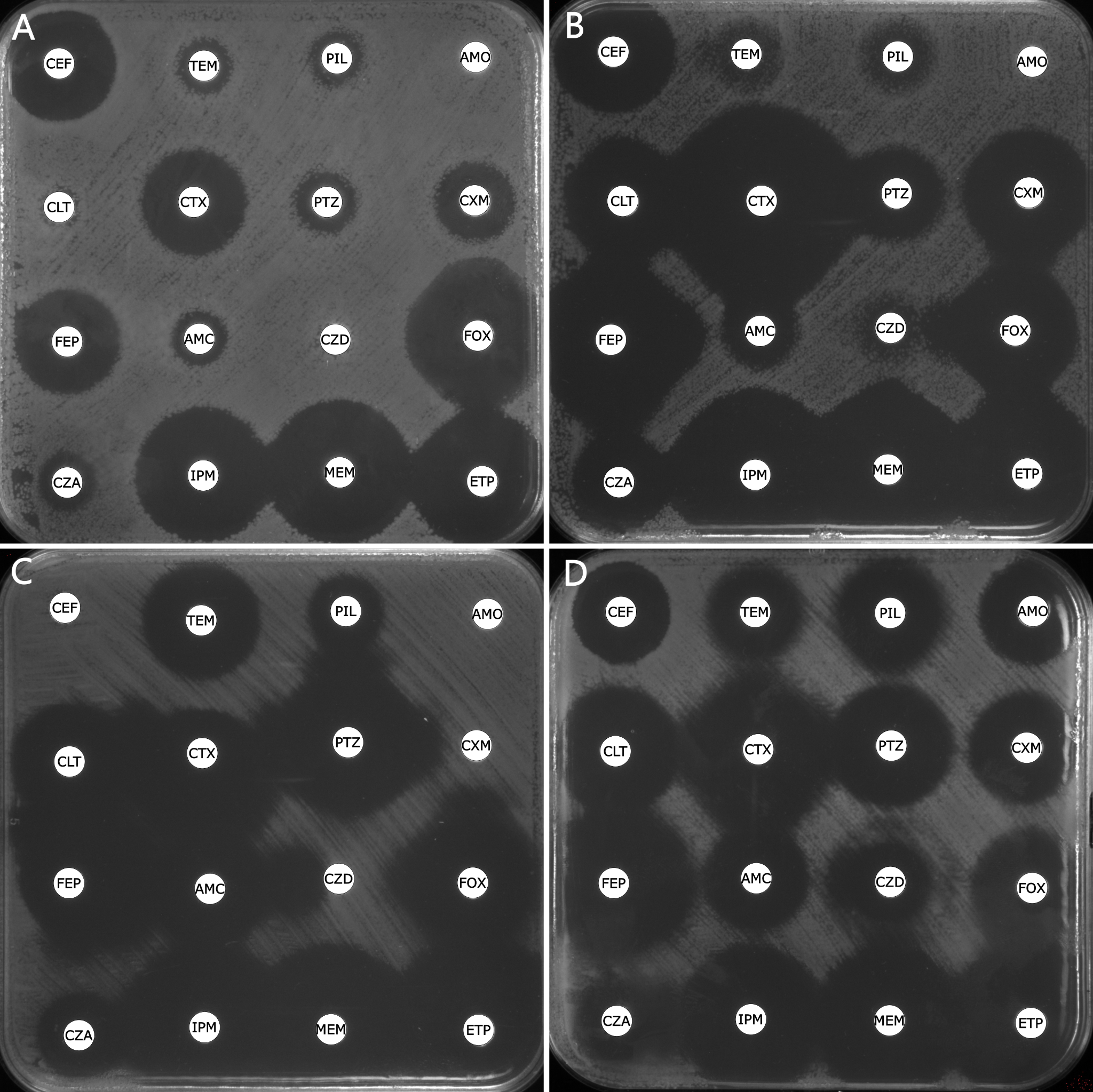


**Figure S3.** Disc diffusion assay for each clone associated with increased cefiderocol MIC reveals diverse ꞵ-lactam resistance phenotypes. **A.** SWE-1-JRYAIN; **B.** GER-3-ELBEWATER; **C.** GER-1-KREISCHAIN; **D.** GER-5-KREISCHAOUT; CEF: cephalotin (30 µg); TEM: temocillin (30 µg); PIL: piperacillin (30 µg); AMO: amoxicillin (20 µg); CLT: ceftolozane (30 µg) + tazobactam (10 µg); CTX: cefotaxime (5 µg); PTZ: piperacillin (30 µg) + tazobactam (6 µg); CXM: cefuroxime (30 µg); FEP: cefepime (30 µg); AMC: amoxicillin (20 µg) + clavulanic acid (10 µg); CZD: ceftazidime (10 µg); FOX: cefoxitine (30 µg); CZA: ceftazidime (10 µg) + avibactam (4 µg); IPM: imipenem (10 µg); MEM: meropenem (10 µg); ETP: ertapenem (10 µg).


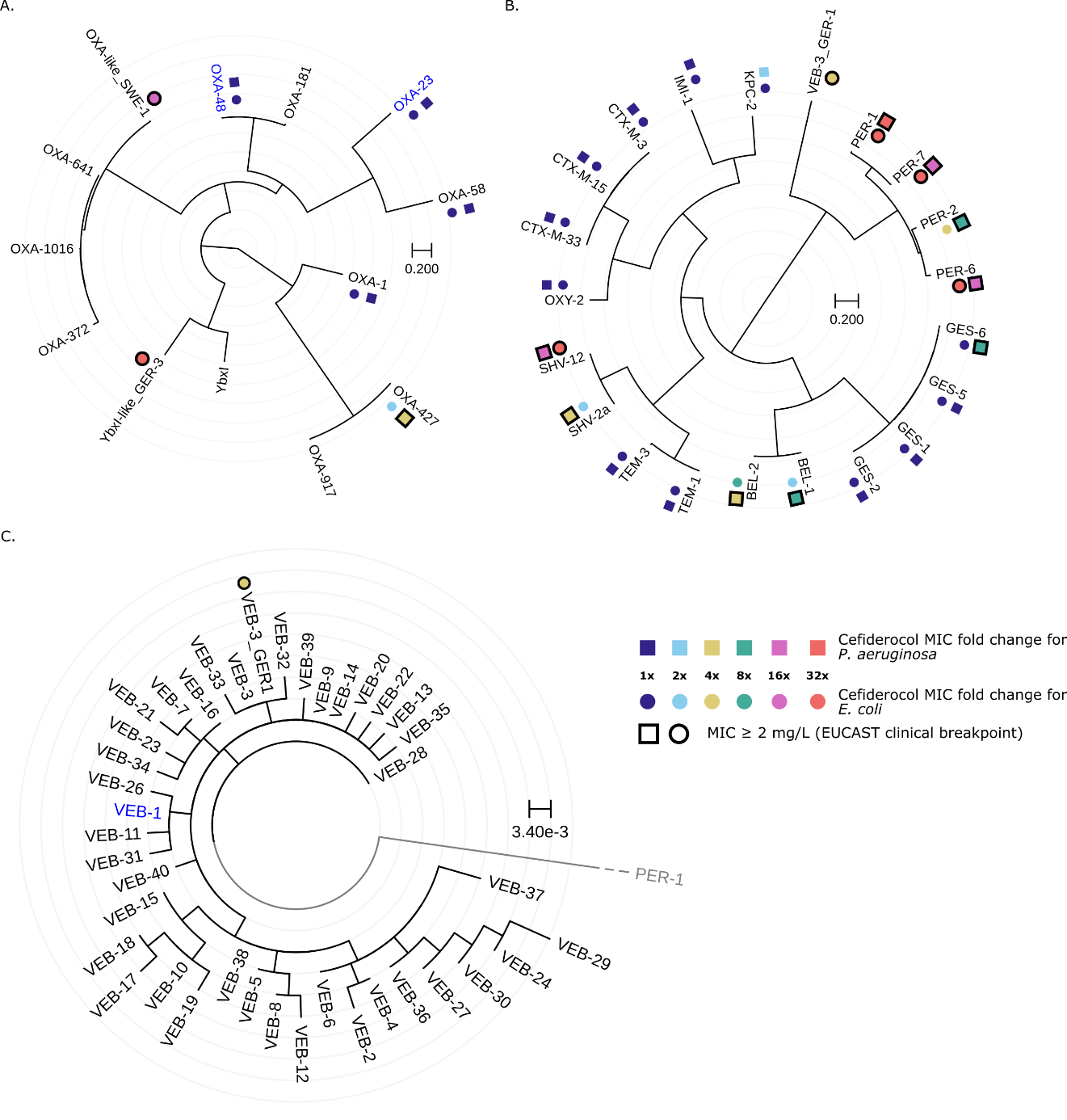


**Figure S4.** **A.** Unrooted phylogenetic tree for Ambler Class D ꞵ-lactamases with available data on associated cefiderocol MICs [[5]](https://www.zotero.org/google-docs/?6TjBlZ). Other close relatives (OXA-181, OXA-372, OXA-641, OXA-917, OXA-1016, and YbxI) were added for context. **B.** Unrooted phylogenetic tree for Ambler Class A ꞵ-lactamases with available cloning MIC increase [[5]](https://www.zotero.org/google-docs/?yNZkCJ). **C.** Circular phylogenetic tree constructed from the amino acid sequences of 39 VEBs (including some with identical sequences, which results in null cophenetic distances) and PER-1 as an outgroup. The alternating gray and white disc-shaped areas correspond to differences of a single amino acid (substitution rate of 3.4E-3), except for the central white disc which represents a null phylogenetic distance. Blue: the beta-lactamase was shown not to hydrolyze cefiderocol; grey: branch artificially shortened.

**
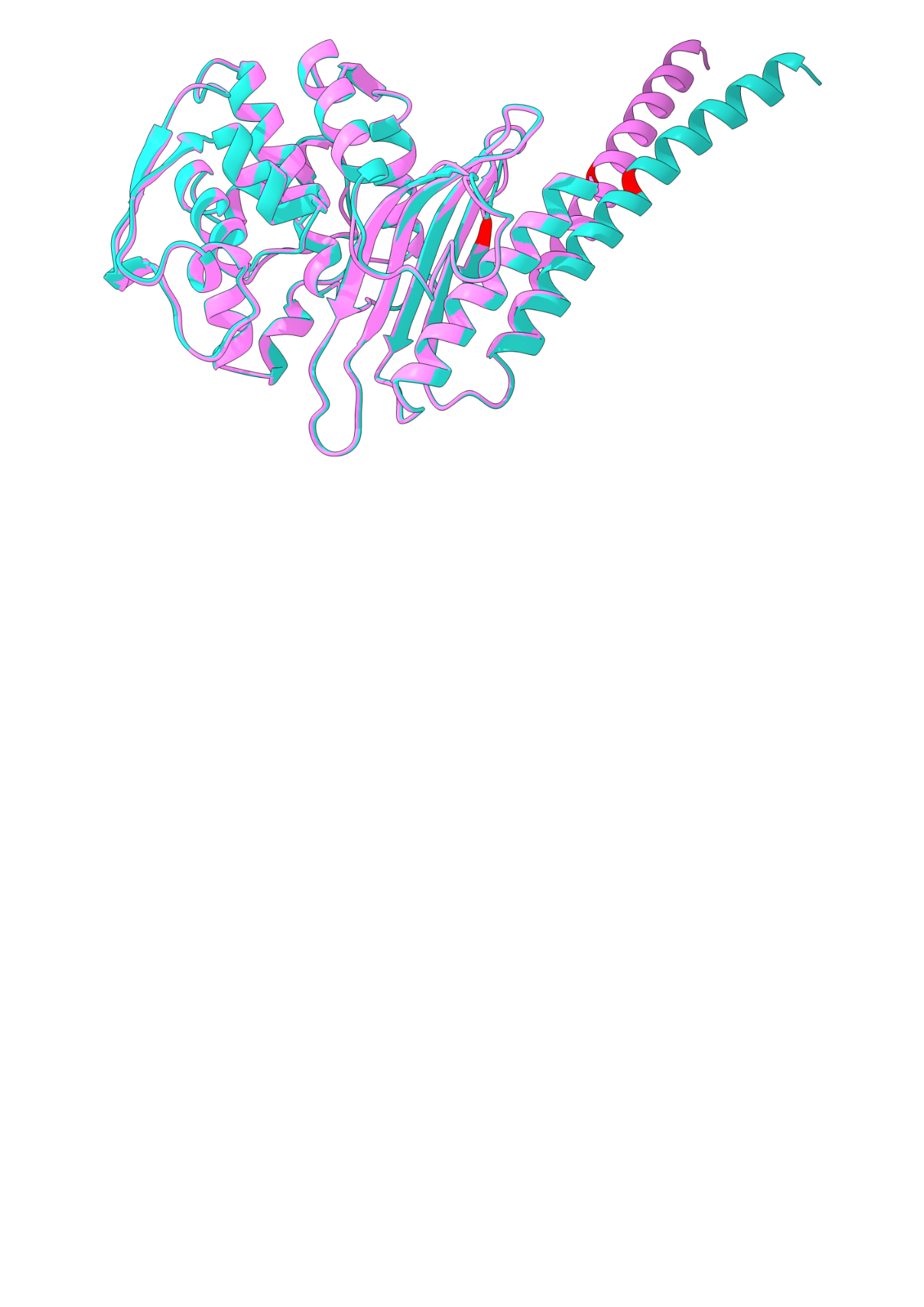
**

**Figure S5.** Structure of unbound VEB-1 (pink) and VEB-3 (cyan) ꞵ-lactamases aligned using the Matchmaker function of ChimeraX software. Amino acid substitutions are highlighted in red.


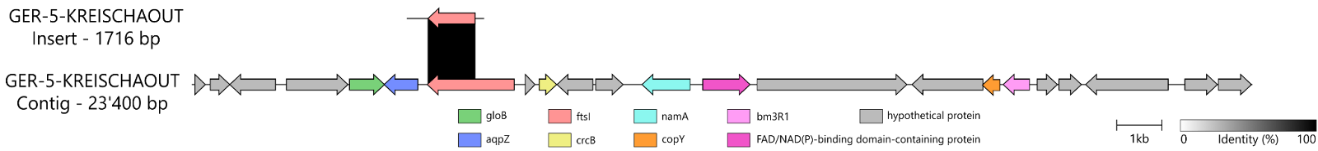


**Figure S6.** Cartography of the insert sequence identified in the GER-5-KREISCHAOUT sample and of the contig from metagenomic sequencing reads assembly of the same sample where the gene could also be found.

**References**

[1.](https://www.zotero.org/google-docs/?yO5OdV)  [Tskhay F et al. Fish are poor sentinels for surveillance of riverine antimicrobial resistance. *One Health* 2025;**20**:101026. https://doi.org/10.1016/j.onehlt.2025.101026](https://www.zotero.org/google-docs/?yO5OdV)

[2.](https://www.zotero.org/google-docs/?yO5OdV)  [Simner PJ, Patel R. Cefiderocol antimicrobial susceptibility testing considerations: The Achilles’ heel of the trojan horse?](https://www.zotero.org/google-docs/?yO5OdV)  *J Clin Microbiol* [2020;**59**:10.1128/jcm.00951-20. https://doi.org/10.1128/jcm.00951-20](https://www.zotero.org/google-docs/?yO5OdV)

[3.](https://www.zotero.org/google-docs/?yO5OdV)  [Prjibelski A et al. Using SPAdes *de novo* assembler.](https://www.zotero.org/google-docs/?yO5OdV)  *Curr Protoc Bioinformatics* [2020;**70**:e102. https://doi.org/10.1002/cpbi.102](https://www.zotero.org/google-docs/?yO5OdV)

[4.](https://www.zotero.org/google-docs/?yO5OdV)  Kille B et al.  [Parsnp 2.0: scalable core-genome alignment for massive microbial datasets. *Bioinformatics* 2024; 40(5):btae311. doi: 10.1093/bioinformatics/btae311.](https://www.zotero.org/google-docs/?yO5OdV)

[5.](https://www.zotero.org/google-docs/?yO5OdV)  [Poirel L et al. Impact of acquired broad-spectrum β-lactamases on susceptibility to cefiderocol and newly developed β-lactam/β-lactamase inhibitor combinations in *Escherichia coli* and *Pseudomonas aeruginosa*. *Antimicrob Agents Chemother* 2022;**66**:e0003922. https://doi.org/10.1128/aac.00039-22](https://www.zotero.org/google-docs/?yO5OdV)

[6.](https://www.zotero.org/google-docs/?yO5OdV)  [Minh BQ et al. QMaker: Fast and accurate method to estimate empirical models of protein evolution. *Syst Biol* 2021;**70**:1046–1060. https://doi.org/10.1093/sysbio/syab010](https://www.zotero.org/google-docs/?yO5OdV)
